# A dynamic nomogram for predicting postoperative nausea and vomiting after laparoscopic surgery: a prospective study

**DOI:** 10.1186/s12871-026-03740-z

**Published:** 2026-03-09

**Authors:** Yufan Lu, Xuezheng Lin, Beiyan Ruan, Shu Liang, Ying Wang, Lin Wang

**Affiliations:** Department of Anesthesia Surgery, Taizhou Central Hospital (Taizhou University Hospital), Zhejiang, China

**Keywords:** Postoperative nausea and vomiting, Dynamic nomogram, Laparoscopic surgery, Prediction model, Logistic regression

## Abstract

**Background:**

The objective of this study was to develop and validate a dynamic nomogram for predicting postoperative nausea and vomiting (PONV) following laparoscopic procedures.

**Methods:**

Clinical data were prospectively collected from adult patients undergoing laparoscopic procedures between March 10, 2025, and May 22, 2025. Patient demographics and clinical characteristics were used to develop a dynamic nomogram for predicting PONV. Variable screening and predictor selection were performed using least absolute shrinkage and selection operator (LASSO) regression, followed by refinement through multivariable logistic regression to construct the nomogram. The area under the curve (AUC) was used to objectively quantify the discriminative ability of the model. Internal validation was performed using bootstrapping, and model performance was further evaluated using calibration and decision curve analysis (DCA).

**Results:**

Of the 413 patients enrolled, 127 (30.8%) developed PONV within 24 h postoperatively. A nomogram incorporating six predictors was developed. The AUC of the prediction model was 0.704 (95% confidence interval [CI]: 0.648‒0.759), and internal validation using bootstrapping was 0.728 (95% CI: 0.674‒0.782). The model demonstrated good calibration, and the DCA revealed a satisfactory net benefit for patients when the probability threshold ranged from 0.12 to 0.54. This indicates the model’s clinical utility for supporting personalized decision-making.

**Conclusions:**

We developed a dynamic nomogram for PONV risk prediction in laparoscopic surgery, demonstrating its adequate performance and providing intuitive clinical decision support.

**Trial registration:**

The trial was registered in the Chinese Clinical Trial Registry (Registration No: ChiCTR2500098281; Date: March 05, 2025).

**Supplementary Information:**

The online version contains supplementary material available at 10.1186/s12871-026-03740-z.

## Background

Postoperative nausea and vomiting (PONV) is one of the most frequent adverse effects observed within the first 24 h post-surgery [1]. Accumulated evidence indicates that the overall incidence of PONV is approximately 30% [2–5]. In recent years, laparoscopic surgery (LS), including hepatic resection and cholecystectomy, has gained widespread clinical adoption owing to its minimally invasive nature. Compared with conventional open surgery, LS offers considerable advantages, including reduced postoperative pain, accelerated recovery, and improved quality of life [6]. However, 40–77% of patients develop PONV after LS [7–9]. This considerable variability can be attributed to differences in specific surgical procedures (e.g., cholecystectomy versus gynecological surgery), duration of pneumoperitoneum, and patient-specific risk factors. PONV substantially reduces patient satisfaction [10, 11] and may increase the risk of serious postoperative complications, including anastomotic leakage, gastroesophageal reflux, and incisional hernia [4]. These sequelae frequently lead to prolonged hospitalization and increased healthcare costs [12]. Effective risk assessment is the first step in the management [13, 14].

The Apfel score [15] remains the most widely used clinical tool to evaluate the risk of PONV. However, its clinical applicability and reliability are limited by variations in surgical procedures, heterogeneous patient populations, and the evolution of PONV management protocols. Multiple studies have reported suboptimal performance in area under the curve (AUC) values when the Apfel score was applied to predict PONV in different surgical types and populations [16, 17]. Stoops et al. [18] advocated for the development of population-specific PONV risk scoring systems to enhance the clinical utility and predictive accuracy.

Despite the development of sophisticated machine learning models for predicting PONV after LS [19, 20], their clinical adoption remains limited. A key constraint is the ‘black-box’ nature of many algorithms, which fail to provide the transparent, intuitive explanations required for clinical trust. In contrast, traditional static nomograms offer interpretability but are constrained by their fixed graphical layout. Dynamic nomograms overcome this limitation by not only providing the intuitive interpretability of static nomograms but also offering the crucial advantage of generating instant, personalized risk estimates through an interactive digital interface, thereby enhancing practical utility in clinical settings.

Research on PONV risk assessment specific to LS remains relatively limited. To address this gap, we conducted a prospective study to develop and validate a dynamic nomogram. We hypothesized that a robust model incorporating established risk factors (e.g., female sex, non-smoking status, history of motion sickness of PONV) [21, 22] could provide personalized risk estimation to inform antiemetic prophylaxis decisions.

## Materials and methods

This study generally adheres to the transparent reporting of a multivariable prediction model for individual prognosis or diagnosis (TRIPOD) [23].

### Study design and participants

**Fig. 1:**
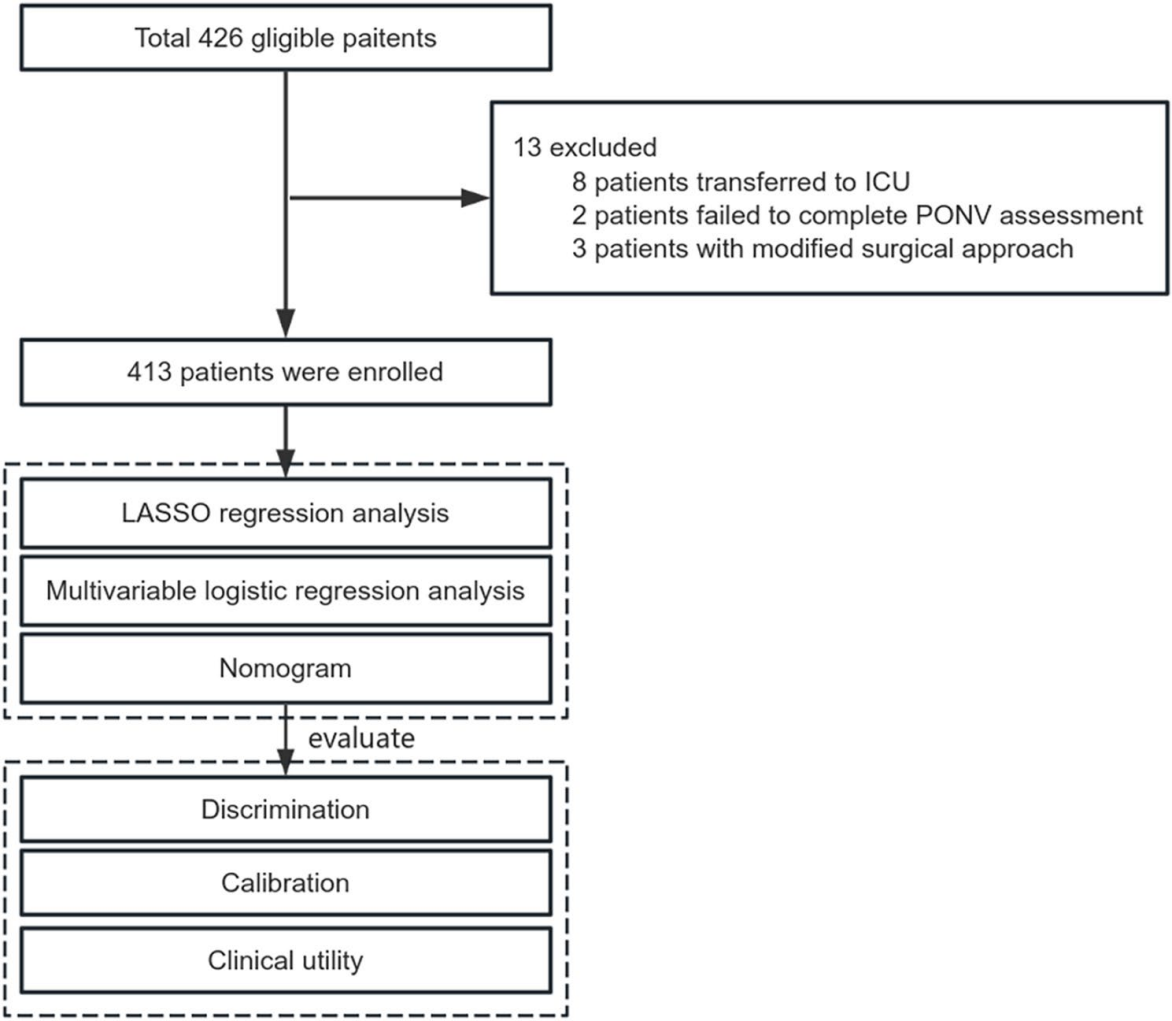
Flowchart of this study. ICU, Intensive care unit; PONV, postoperative Nausea and Vomiting; LASSO, least absolute shrinkage and selection operator

Data were prospectively collected from patients undergoing LS between March 10, 2025, and May 22, 2025 at a Chinese teaching tertiary hospital. The inclusion criteria were as follows: patients aged ≥ 18 years who underwent elective LS. The exclusion criteria were as follows: patients whose surgical method was changed to open surgery; patients with a history of severe mental illness or impaired normal communication capacity; patients who refused to sign the informed consent form; patients with incomplete medical records or those who failed to complete the PONV assessment; and patients transferred to the intensive care unit post-surgery. Figure 1 illustrates a flowchart of the study.

### Definition of outcome variables

The primary outcome was the incidence of PONV, as determined by postoperative patient follow-up. The diagnostic criterion for PONV was nausea or vomiting within 24 h postoperatively. Nausea was defined as a subjectively unpleasant sensation accompanied by a conscious urge to vomit. Vomiting was characterized as either productive (successful expulsion) or nonproductive (retching) ejection of gastric contents. Patients were stratified into PONV and non-PONV groups according to symptom occurrence within 24 h postoperatively.

### Anesthesia protocol

All patients underwent routine intraoperative monitoring, including noninvasive blood pressure monitoring and electrocardiography. A combined intravenous general anesthesia protocol was used, consisting of sufentanil (0.6 µg/kg), propofol (2 mg/kg), and rocuronium (0.6 mg/kg) for induction. Sevoflurane, with or without remifentanil, was used for intraoperative maintenance. Rocuronium was administered intermittently during the surgery to maintain muscle relaxation. The depth of anesthesia was adjusted to maintain the bispectral index value within the target range of 40‒60. Vasoactive drugs were administered, or the infusion plan was adjusted as needed to maintain hemodynamic stability.

The intraoperative multimodal antiemetic regimen was not standardized. The decision to administer prophylaxis, as well as the selection of specific agents (such as a single intraoperative dose of dexamethasone 5–10 mg and/or ondansetron 4–8 mg), was made at the discretion of the attending anesthesiologist based on clinical judgment. Sugammadex was administered to antagonize postoperative residual muscle relaxation. Once extubation criteria were met, patients were extubated in the operating theater and transferred to the post-anesthesia care unit for at least 30 min of monitoring. Postoperative analgesia was tailored to the patient’s preference, and a patient-controlled analgesia (PCA) regimen, mainly based on sufentanil, was used.

### Data collection

Through a systematic review of the existing literature, we collected variables potentially associated with PONV (Table 1) [24–26]. Candidate variables included preoperative and anesthesia-/surgery-related factors.

**Table 1:** Baseline characteristics of patients

Variables	Total (*n* = 413)	PONV (*n* = 127)	Non-PONV (*n* = 286)	*P* value
Female, *n* (%)	211(51.09%)	84(66.14%)	127(44.41%)	< 0.001
Age, year	60 [46;69]	56[44;65]	60 [47;70]	0.007
Height, cm	163[157;169]	160[156;165]	164[158;170]	< 0.001
Weight, kg	63 [55;71]	60[53;68]	65[57;72]	< 0.001
Current smoker, *n* (%)	77 (18.64%)	14 (11.02%)	63 (22.03%)	0.012
History of motion sickness or PONV, *n* (%)	116 (28.09%)	48 (37.80%)	68 (23.78%)	0.005
Hypertension, *n* (%)	116 (28.09%)	28 (22.05%)	88 (30.77%)	0.089
Diabetes mellitus, *n* (%)	41 (9.93%)	5 (3.94%)	36 (12.59%)	0.011
History of migraine, *n* (%)	74 (17.92%)	29 (22.83%)	45 (15.73%)	0.110
Stomach illness, *n* (%)	134 (32.45%)	44 (34.65%)	90 (31.47%)	0.601
Anxiety or depression, *n* (%)	32 (7.75%)	12 (9.45%)	20 (6.99%)	0.508
Fasting time (fluids), h	4.50 [3.00;7.00]	4.00 [3.00;6.00]	4.50 [3.00;7.00]	0.085
Fasting time (solids), h	17.00 [15.00;19.00]	16.50 [14.50;19.00]	17.00 [15.00;19.00]	0.477
ASA class, *n* (%)				0.631
I	95 (23.00%)	33 (25.98%)	62 (21.68%)	
II	301 (72.88%)	89 (70.08%)	212 (74.13%)	
III	17 (4.12%)	5 (3.94%)	12 (4.20%)	
Anesthesiologist experience, *n* (%)				0.485
≤5year	22 (5.33%)	17 (5.94%)	5 (3.94%)	
6-10year	127 (30.75%)	93 (32.52%)	34 (26.77%)	
11-15year	112 (27.12%)	75 (26.22%)	37 (29.13%)	
> 15year	152 (36.80%)	101 (35.31%)	51 (40.16%)	
Type of surgery, *n* (%)				0.191
general surgery	302 (73.12%)	93 (73.23%)	209 (73.08%)	
urology	29 (7.02%)	5 (3.94%)	24 (8.39%)	
gynecology	82 (19.85%)	29 (22.83%)	53 (18.53%)	
Duration of surgery, minute	80.00[60.00;125.00]	80.00[55.00;132.50]	80.00[60.00;125.00]	0.856
Total fluid infusion, ml	1000[500;1000]	1000 [500;1000]	1000[500;1000]	0.736
Sufentanil, µg	30.00 [30.00;50.00]	30.00 [27.50;47.50]	30.00 [30.00;50.00]	0.969
Remifentanil, mg	1.00 [0.50;1.00]	1.00 [0.45;1.00]	1.00 [0.50;1.00]	0.824
Use of dexmedetomidine, *n* (%)	122 (29.54%)	36 (28.35%)	86 (30.07%)	0.812
Use of glucocorticoids, *n* (%)	316 (76.51%)	88 (69.29%)	228 (79.72%)	0.029
Use of ondansetron, *n* (%)	329 (79.66%)	97 (76.38%)	232 (81.12%)	0.331
PCA used after surgery, *n* (%)	36 (8.72%)	10 (7.87%)	26 (9.09%)	0.829

*Abbreviations*: *PONV* postoperative nausea and vomiting, *ASA* American Society of Anesthesiologists, *PCA* patient-controlled analgesia

Preoperative variables included sex, age, height, weight, current smoking status, history of motion sickness or PONV, history of migraine, diabetes mellitus, hypertension, stomach illness (defined as a documented history of gastroesophageal reflux disease, peptic ulcer disease, or chronic gastritis), anxiety or depression, and fasting time (fluids/solids). Anesthesia- and surgery-related variables included anesthesiologist experience (up to 5 years, 6 to 10 years, 11 to 15 years, and over 15 years), American Society of Anesthesiologists (ASA) class, type of surgery, duration of surgery, total fluid infusion, intraoperative medication (remifentanil, sufentanil, glucocorticoids, dexmedetomidine, and ondansetron), and postoperative PCA pump use.

### Sample size

The sample size calculation for this study was based on the events-per-variable (EPV) criterion for logistic regression proposed by Peduzzi et al. [27], which confirms that 6 to 10 events per independent variable are required to ensure model validity. Based on the study design, it was anticipated that the final model would include 8 independent variables; thus, applying the EPV = 10 standard, a minimum of 80 positive events was required. Based on a reported PONV incidence of 40.4% after LS in another study [4], the theoretical minimum sample size was calculated to be 80 ÷ 0.404 ≈ 198 participants. Furthermore, accounting for a 10% attrition rate, the final required sample size was determined as 198 + (198 × 0.10) ≈ 218 participants.

### Statistical analysis

Data analyses were conducted using R software (version 4.4.3) and IBM SPSS Statistics (version 29.0). The dataset was complete with no missing values for any variable. Continuous variables were processed according to their distribution characteristics: data following a normal distribution are presented as the mean and standard deviation, with the t-test employed for between-group comparisons; non-normally distributed data are presented as the median (interquartile range) and analyzed using the Mann-Whitney U test. Categorical variables are expressed as counts (percentages) and compared using the chi-square or Fisher’s exact test, depending on the sample size. Statistical significance was defined as a *p*-value < 0.05.

Multicollinearity was assessed using Tolerance (Tol) and Variance Inflation Factor (VIF), with Tol > 0.1 or VIF < 10 indicating no significant issues. Preliminary variable screening was then conducted using least absolute shrinkage and selection operator (LASSO) regression. A sensitivity analysis was performed to evaluate the stability of variable selection under different penalty parameters, in which the LASSO model was refitted at λ_min, 0.8 × λ_min, and 1.2 × λ_min. Subsequently, variables with nonzero coefficients from the LASSO regression were subjected to multivariable logistic regression.

Variables for the final prediction model were selected using Akaike Information Criterion (AIC)-based backward stepwise regression. Based on the logistic regression results, we developed a dynamic nomogram using the ‘Dynnom’ package, with an interactive web application created via Shiny.

Model discrimination was assessed using AUC values [28], with internal validation performed using 1000 bootstrap replicates to avoid overfitting [23]. Nomogram calibration was assessed graphically and statistically, employing calibration curves and the Hosmer-Lemeshow goodness-of-fit test. A *p*-value > 0.05 indicated a good goodness-of-fit. Finally, the nomogram’s clinical utility was evaluated using decision curve analysis (DCA) [29].

## Results

### Patient characteristics

Perioperative data were prospectively collected for 426 patients aged ≥ 18 years who were scheduled for elective LS at our hospital. Based on the predefined eligibility criteria, 13 patients were excluded from the study cohort. Finally, 413 patients who underwent surgery were included in this study (Fig. 1). Table 1 summarizes the baseline characteristics of patients. A total of 127 patients (30.8%) experienced PONV within 24 h post-surgery. As anticipated, patients who developed PONV were more likely to be female, non-smokers, and have a history of motion sickness or PONV (all *p* < 0.05). They were also younger and had lower body weight and height compared to the non-PONV group (all *p* < 0.01). Further, they showed a lower prevalence of diabetes mellitus and a reduced utilization rate of glucocorticoids (both *p* < 0.05). The remaining variables did not differ significantly between differences (all *p*-values > 0.05).

### Identification of predictive factors

There was no significant multicollinearity among the variables, as evidenced by Tol values all above 0.1 and VIF all well below 10 (Supplementary Table 1).

**Fig. 2:**
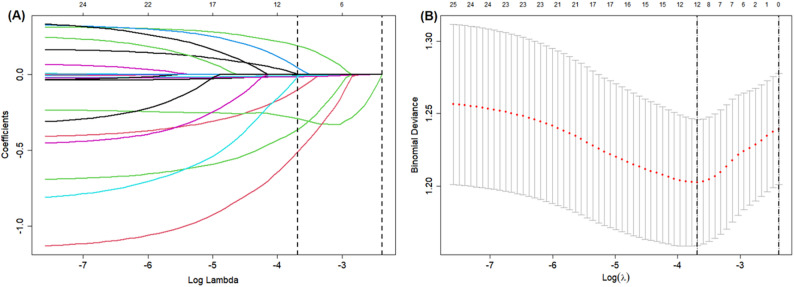
Variable selection via LASSO regression. **A** Coefficient profiles of candidate predictors, demonstrating how each variable’s contribution changes with the penalty parameter (λ). **B** Optimal λ selection using 10-fold cross-validation. Vertical dotted lines indicate λ values chosen by the minimum MSE criterion (left) and the one-standard-error rule (right)

The analysis incorporated 24 predictor variables with PONV incidence as the response variable. Through ten-fold cross-validation, the optimal penalization coefficient λ was determined to be λ_min = 0.02517. Twelve nonzero characteristic variables were selected: anesthesiologist experience, current smoker, history of motion sickness or PONV, history of migraine, fasting time of clear fluids, sex, age, height, weight, diabetes mellitus, use of glucocorticoid, and type of surgery (Fig. 2A and B).

Furthermore, sensitivity analysis confirmed robust variable selection across a range of λ values (0.02014 to 0.03021). The variable set remained identical between λ = 0.02014 and λ = 0.02517; at the more stringent λ = 0.03021, 10 of the 12 variables (83%) were consistently retained (Supplementary Table 2). Notably, multivariable logistic regression analyses were performed separately for both variable sets: one using the 12 variables identified at λ_min = 0.02517, and another using the 10 variables retained at λ = 0.03021. Both analyses converged to the same six predictors of PONV: current smoker, history of motion sickness or PONV, age, weight, diabetes mellitus, and use of glucocorticoid (Table 2). This consistency across λ values and variable sets further validates the stability of the selected core predictors.

**Table 2:** Prediction model with Multivariable logistic regression

Variables	Odds Ratio(95% CI)	*P* value
Current smoker, yes vs. no	0.559 (0.281–1.051)	0.082
History of motion sickness or PONV, yes vs. no	1.504 (0.933–2.413)	0.091
Age, year	0.983 (0.968–0.999)	0.039
Weight, kg	0.965 (0.945–0.985)	< 0.001
Diabetes mellitus, yes vs. no	0.341 (0.110–0.864)	0.037
Use of glucocorticoid, yes vs. no	0.492 (0.296–0.816)	0.006

*Abbreviations*: *PONV* postoperative nausea and vomiting

### Construction of a dynamic nomogram model for predicting PONV

**Fig. 3:**
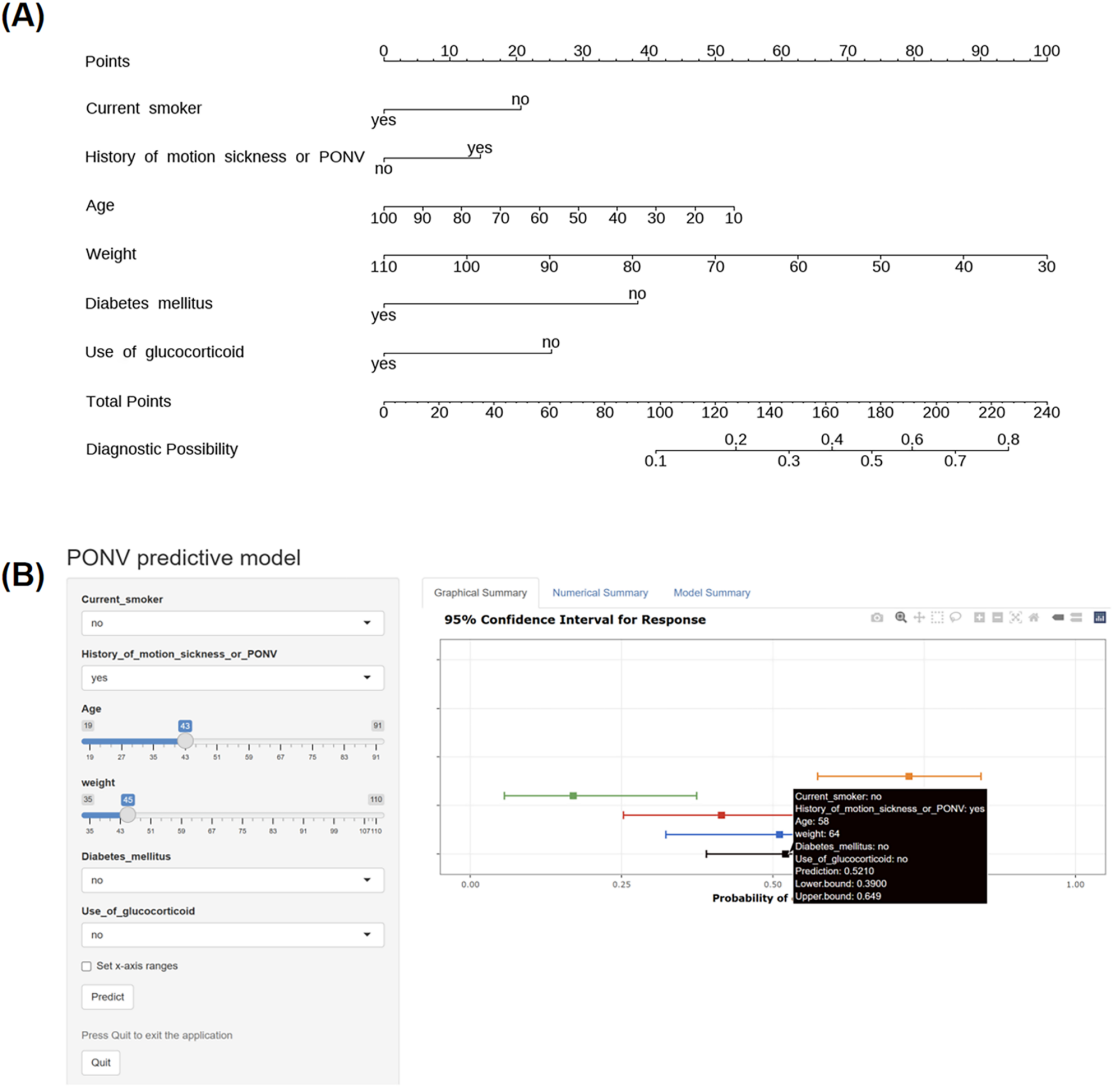
Nomograms for predicting postoperative nausea and vomiting (PONV) risk. **A** Conventional static nomogram demonstrating the scoring system for predicting PONV risk. Each predictor variable was assigned a point scale, with the total points corresponding to the predicted probability on the final axis. **B** Interactive dynamic nomogram providing real-time probability estimates with 95% confidence intervals. The “Numerical Summary” tab displays: (1) user-input clinical parameters, (2) calculated risk probability, and (3) associated confidence bounds for clinical decision-making

A clinical prediction nomogram was derived from the multivariable logistic regression model incorporating six predictors to visualize the predicted risk of PONV. First, we developed a traditional static nomogram model by assigning corresponding scores to each predictive variable and predicting the probability of a target event by calculating the total score (Fig. 3A). However, considering the limitations of static nomograms in clinical practice (e.g., subjective interpretation and insufficient adaptability to complex models), we developed an interactive dynamic nomogram using the Shiny framework to enhance user accessibility. This online tool enables clinicians to input relevant values of each predictive variable for patients through the interface, with the system automatically calculating the predictive probability in real time and generating visualized results, including graphical risk displays and precise numerical summaries (Fig. 3B). The dynamic nomogram is readily accessible to all users via a dedicated web interface (https://luyufan9256.shinyapps.io/DynNomapp/), requiring no specialized software installation.

### Performance of the dynamic nomogram

**Fig. 4:**
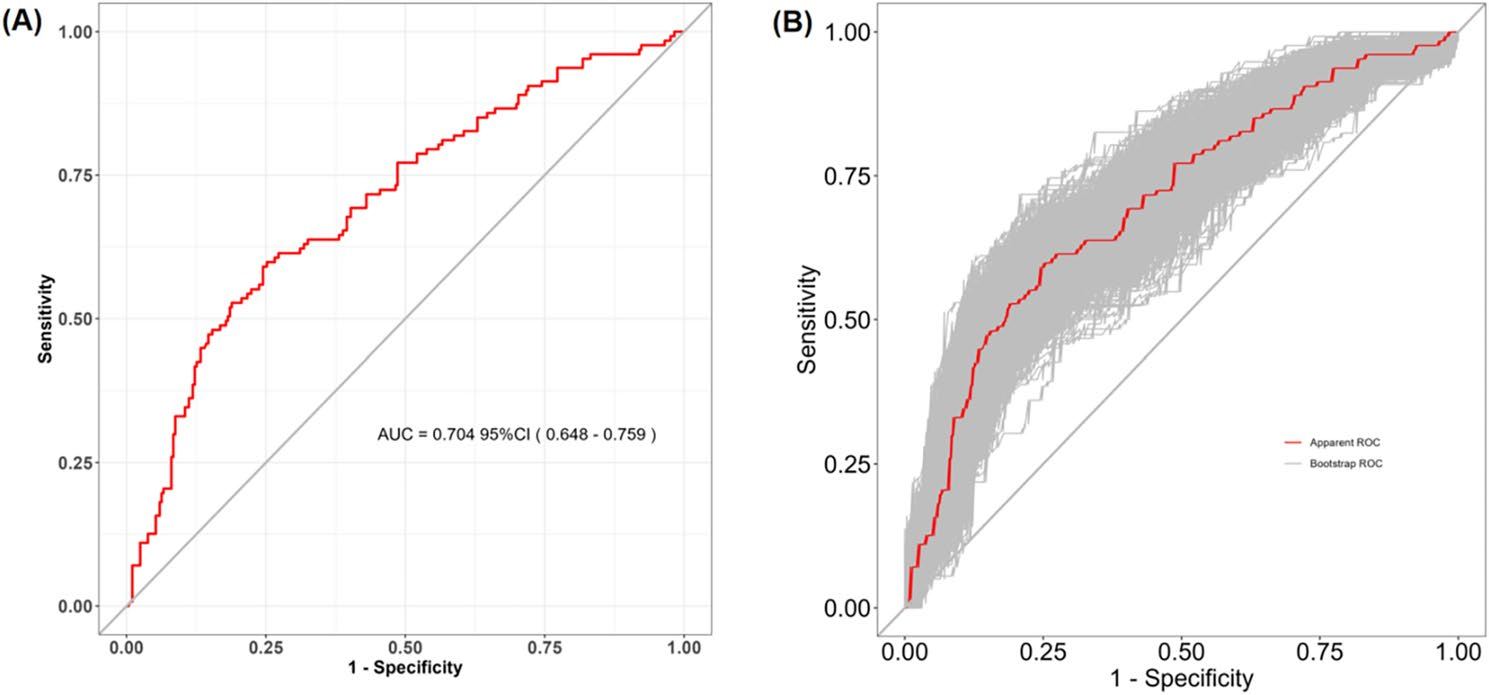
AUC and internal validation. **A** ROC curve showing the apparent performance of the prediction model (AUC = 0.704; 95% CI: 0.648‒0.759). **B** Bias-corrected ROC curve derived from 1000 bootstrap resamples (AUC = 0.728; 95% CI: 0.674‒0.782). The gray shaded region represents the 95% CI of the ROC curve from the bootstrap validation. AUC, area under the curve; CI, confidence interval; ROC, receiver operating characteristic

**Fig. 5:**
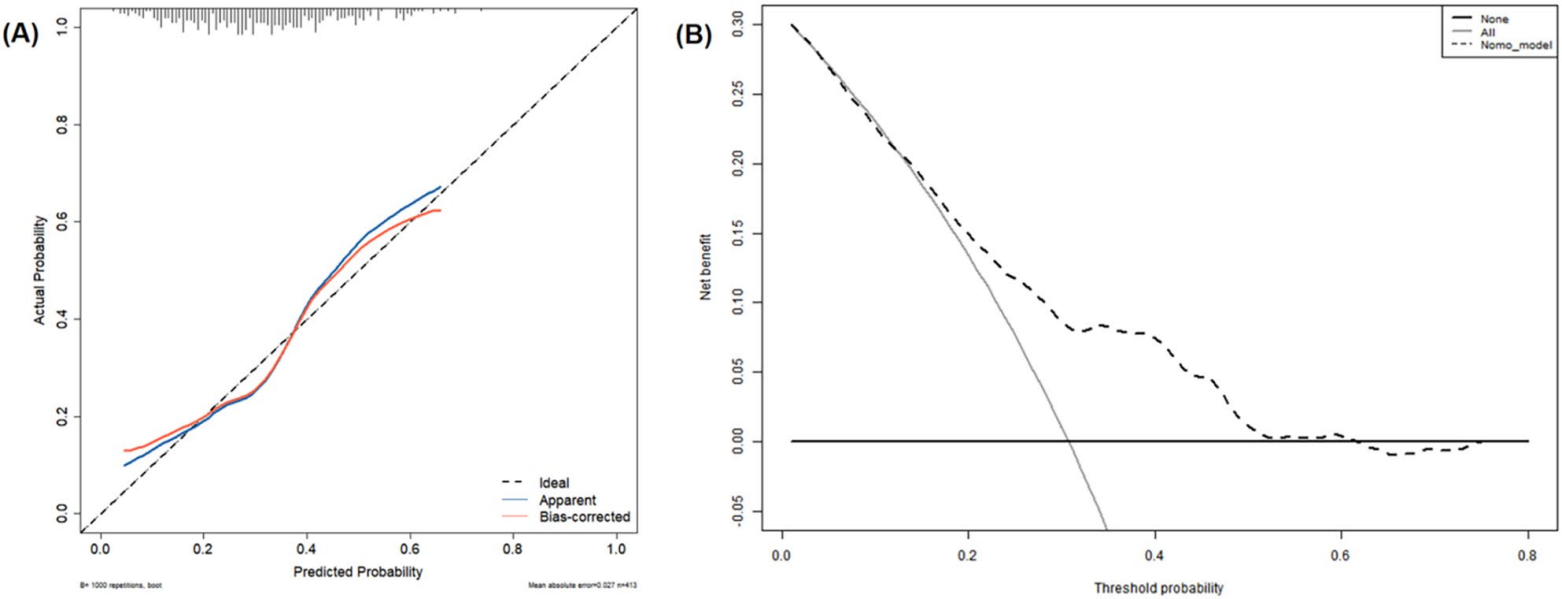
Calibration plot and decision curve analysis. **A** Calibration plot of the prediction model (1000 bootstrap resamples). The dashed line is ideal calibration. The blue solid line shows apparent calibration, and the red solid line shows bias-corrected calibration. **B** Decision curve analysis. The dashed black line indicates the net benefit of using the nomogram to guide antiemetic prophylaxis. The black solid line represents treating no patients, and the gray solid line represents treating all patients. The nomogram is clinically useful where the dashed black line exceeds the reference lines

The AUC of the prediction model reached 0.704 (95% confidence interval [CI]: 0.648‒0.759) (Fig. 4A). As illustrated in Fig. 4B, internal validation using the bootstrapping method (resampling = 1000) yielded an AUC of 0.728 (95% CI: 0.674‒0.782). Internal validation was performed by bootstrapping with 1000 replicates of the original dataset to generate the calibration curves. Figure 5A presents the calibration plot, revealing a close alignment between the model predictions and observed outcomes. The Hosmer-Lemeshow test supported this finding (χ² = 6.8976, *p* = 0.5477), indicating satisfactory model calibration. DCA was conducted to evaluate the clinical utility of the model. The DCA curve indicated that, across a threshold probability range of 12% to 54%, using the nomogram to guide decisions provided a greater net benefit than the strategies of treating all or no patients (Fig. 5B).

## Discussion

PONV is a critical clinical issue that affects postoperative recovery and may increase the risk of aspiration, potentially endangering the patients’ lives. In the current study, PONV occurred in 30.8% of the 413 enrolled patients, which is consistent with previously reported data [2–4]. Timely and accurate identification of patients at risk for PONV enables targeted prophylaxis, including the rational use of antiemetic medications, adjustment of opioid dosages, and provision of adjuvant support. These measures can enhance postoperative recovery rates, thereby effectively improving patient satisfaction. In recent years, machine learning has been applied to PONV risk prediction. One retrospective analysis of 106,860 patients across two hospitals developed models via logistic regression, random forest, light gradient boosting, and multi-layer perceptron, with AUCs 0.60‒0.67 [30]. Another study of 35,003 adult inpatients created an ensemble model combining multiple machine learning algorithms, demonstrating strong discrimination: AUC 0.872 for early PONV and 0.708 for delayed PONV [31]. However, these models target broad surgical populations (not specifically LS) and their complex algorithms limit interpretability. To address this gap, we developed a dynamic nomogram specifically for LS patients.

To enable precise, individualized risk estimation beyond categorical risk stratification, our model integrates six predictors into a visual, web-based interface for objective and convenient PONV risk prediction. Internal validation suggested promising discriminative ability and calibration in our cohort of patients undergoing LS. DCA demonstrated that for any clinician whose intervention threshold falls within the 12%–54% range, using this nomogram to guide treatment (e.g., initiating an enhanced antiemetic regimen for patients with a predicted risk > 20%) yields a net benefit. This supports the optimization of clinical decision-making by guiding enhanced prophylaxis for high-risk patients while avoiding overtreatment in low-risk populations. Furthermore, compared with traditional nomograms, it offers greater clinical applicability and convenience, supporting the model’s widespread promotion. To ensure practical implementation across diverse settings, we provide both an interactive web tool and a static nomogram in Fig. 3, the latter enabling offline use in resource-limited environments.

The variables in our final prediction model were selected based on their collective contribution to model performance via an AIC-based algorithm, rather than on individual statistical significance alone. Accordingly, the model retained smoking status and a history of motion sickness or PONV. Although their individual *p*-values exceeded 0.05, their well-established association with PONV in numerous studies [18, 32, 33] provides a strong rationale for their inclusion. For instance, a history of motion sickness or PONV is linked to heightened emetic reflex pathway activity and a lowered PONV threshold [18], while nicotine from smoking inhibits 5-HT_3_ receptor function [34]. Their retention likely enhances the model’s discriminant ability and clinical applicability by ensuring it reflects recognized risk profiles.

Among the predictors in our model with stronger statistical associations, younger age was significantly associated with an increased risk of PONV, consistent with findings from a meta-analysis by Apfel et al. [33]. The incidence of PONV decreases with increasing age, which may be related to the attenuation of age-associated autonomic reflexes that trigger nausea or vomiting [33].

Notably, our prediction model identified an inverse association between diabetes status and PONV risk in patients undergoing LS. Evidence regarding the impact of diabetes-related characteristics on PONV remains limited and controversial. For example, Ding et al. [35] evaluated 334 patients undergoing laparoscopic bariatric surgery and reported a statistically significant reduction in the incidence of PONV among patients with diabetes compared with their non-diabetic counterparts. This result is consistent with the findings of the present study, suggesting that the diabetes status may influence PONV occurrence through certain pathophysiological mechanisms. However, a retrospective cohort study reported different conclusions [36]. The study compared complications after bariatric surgery between patients with and without diabetes and reported similar incidence rates of immediate postoperative PONV in both groups (8.7% in the non-diabetic group vs. 9.0% in the diabetic group). These discrepancies in the results may be attributed to inconsistencies in patient populations, diabetes characteristics, and perioperative management. Future prospective studies with larger sample sizes that incorporate diabetes duration, glycemic control, and gastrointestinal function assessment are needed to further clarify the relationship between diabetes mellitus and PONV.

Furthermore, use of glucocorticoids was associated with a reduced risk of PONV in our prediction model. This outcome aligns with previous studies [37, 38], suggesting that this variable is a robust component in predictive algorithms for PONV. Glucocorticoids may exert their anti-PONV effects through multiple mechanisms, including anti-inflammatory actions; direct central effects on the nucleus of the solitary tract; interactions with neurotransmitters such as serotonin and receptor proteins including tachykinin NK1, NK2, and alpha-adrenergic receptors; maintenance of normal physiological functions of organs and systems; regulation of the hypothalamic-pituitary-adrenal axis; and reduction of pain and concomitant opioid use, which in turn decrease opioid-related nausea and vomiting [39].

In this study, higher body weight was identified as being associated with a lower risk of PONV. Consistent with Poon et al. [40], each 1-kg increase in body weight correlated with a 2% decrease in the incidence of PONV. This association is also supported by a recent machine learning-based study, which ranked body weight among the top 10 most important predictors of PONV and noted that patients with lower body weight were more prone to PONV [41]. However, the exact mechanism underlying this observed association remains incompletely understood. Further research is warranted, focusing on physiological pathways (e.g., differences in drug metabolism or neuroendocrine regulation between different weight groups) to clarify this association.

Whilst the model demonstrates clinical utility and acceptable discriminatory performance (AUC = 0.704) for predicting PONV, the factors limiting its performance require further investigation. A key limitation lies in unmeasured genetic variability. For instance, a systematic review on PONV and genetic variability identified two single nucleotide polymorphisms—CHRM3 rs2165870 and KCNB2 rs349358—reported to exert a major influence on PONV incidence [42]. Our study did not incorporate these genetic factors. The absence of such genetic data, coupled with residual clinical confounders (e.g., intraoperative anesthetic management) and unmeasured psychosocial factors, likely constrained the model’s discriminative power. Future research integrating genomic profiling, detailed intraoperative data collection, and psychometric assessments could address these gaps and further improve predictive accuracy.

This study has several other limitations. First, the generalizability of our single-center model remains a primary limitation. Therefore, external validation in diverse, multi-center cohorts is an essential prerequisite and represents the indispensable next step before this nomogram can be considered for routine clinical adoption. Second, the set of predictors considered may be incomplete, potentially omitting certain variables relevant to PONV—including the aforementioned genetic variability—and future work will expand data collection to address this. Third, while surgery was categorized by discipline, we did not separately analyze procedure-specific risks (e.g., cholecystectomy vs. hepatic resection) due to sample size limitations. Future research with larger cohorts should explore such granular distinctions.

## Conclusions

This study presents a dynamic nomogram to predict the risk of PONV in patients undergoing LS. The model incorporates six key predictive variables and achieves an AUC of 0.704. This tool demonstrates good clinical applicability for individualized risk assessment. Nevertheless, further validation through multicenter studies is warranted to confirm the generalizability of our results.

## Supplementary Information

Supplementary Material 1.

## Data Availability

The raw data supporting the findings of this study are available from the corresponding author upon request.

## Abbreviations

PONV: Postoperative nausea and vomiting
LS: Laparoscopic surgery
AUC: Area under the curve
PCA: Patient-controlled analgesia
ASA: American Society of Anesthesiologists
LASSO: Least absolute shrinkage and selection operator
AIC: Akaike Information Criterion
DCA: Decision curve analysis
EPV: events-per-variable
Tol: Tolerance
VIF: Variance Inflation Factor
ROC: Receiver operating characteristic
