# Supplementary material for: A dynamic nomogram for predicting postoperative nausea and vomiting after laparoscopic surgery: a prospective study
Source: BMC Anesthesiol. 2026 Mar 9;26:240. doi: 10.1186/s12871-026-03740-z (PMC13085308; doi:10.1186/s12871-026-03740-z)
Supplement: Supplementary file 1 — Supplementary Material 1. [file 12871_2026_3740_MOESM1_ESM.docx]

**Supplementary Table 1** Collinearity analysis of all related variables

| Collinear statistics | | |
| --- | --- | --- |
| Variables | Tol | VIF |
| Anesthesiologist experience class | 0.838 | 1.193 |
| Current smoker | 0.749 | 1.335 |
| History of motion sickness or PONV | 0.856 | 1.168 |
| History of migraine | 0.921 | 1.086 |
| Stomach illness | 0.940 | 1.064 |
| Anxiety or depression | 0.904 | 1.106 |
| Fasting time fluids | 0.814 | 1.228 |
| Fasting time solids | 0.814 | 1.229 |
| Sex | 0.341 | 2.934 |
| Age | 0.541 | 1.849 |
| height | 0.375 | 2.665 |
| Weight | 0.546 | 1.830 |
| Hypertension | 0.721 | 1.387 |
| Diabetes mellitus | 0.840 | 1.190 |
| ASA class | 0.681 | 1.468 |
| Duration of surgery | 0.440 | 2.271 |
| Total fuid infusion | 0.422 | 2.371 |
| Sufentanil | 0.866 | 1.155 |
| Remifentanil | 0.709 | 1.411 |
| Use of dexmedetomidine | 0.851 | 1.175 |
| Use of glucocorticoid | 0.698 | 1.432 |
| ondansetron | 0.766 | 1.305 |
| Type of surgery | 0.686 | 1.458 |
| PCA used after surgery | 0.654 | 1.529 |

Abbreviations: ASA, American Society of Anesthesiologists; PCA, patient-controlled analgesia; PONV, postoperative nausea and vomiting; Tol, Tolerance; VIF, Variance inflation factor.

****Supplementary Table 2** Sensitivity Analysis of Variable Selection Across Different λ Values**

| Variable | λ = 0.02014 | λ = 0.02517 | λ = 0.03021 | Selection Frequency |
| --- | --- | --- | --- | --- |
| Current smoker | ✓ | ✓ | ✓ | 3/3 |
| History of motion sickness or PONV | ✓ | ✓ | ✓ | 3/3 |
| Age | ✓ | ✓ | ✓ | 3/3 |
| Weight | ✓ | ✓ | ✓ | 3/3 |
| Diabetes mellitus | ✓ | ✓ | ✓ | 3/3 |
| Use of glucocorticoid | ✓ | ✓ | ✓ | 3/3 |
| History of migraine | ✓ | ✓ | ✓ | 3/3 |
| Fasting time fluids | ✓ | ✓ | ✓ | 3/3 |
| Sex | ✓ | ✓ | ✓ | 3/3 |
| height | ✓ | ✓ | ✓ | 3/3 |
| Anesthesiologist experience class | ✓ | ✓ | - | 2/3 |
| Type of surgery2 | ✓ | ✓ | - | 2/3 |
| **Total Variables Selected** | **12** | **12** | **10** | ****-**** |

Abbreviations: PONV, postoperative nausea and vomiting.

✓ indicates the variable was selected at the given λ value. The selection frequency represents the number of models (out of three: λ = 0.02014, 0.02517, and 0.03021) in which each variable was selected. λ = 0.09259 excluded all variables and is therefore not included in the frequency calculation.
